# Are Plasma Biomarkers of Immune Activation Predictive of HIV Progression: A Longitudinal Comparison and Analyses in HIV-1 and HIV-2 Infections?

**DOI:** 10.1371/journal.pone.0044411

**Published:** 2012-09-10

**Authors:** Samuel Nyamweya, John Townend, Akram Zaman, Sarah Jane Steele, David Jeffries, Sarah Rowland-Jones, Hilton Whittle, Katie L. Flanagan, Assan Jaye

**Affiliations:** 1 Medical Research Council (UK), Banjul, The Gambia, West Africa; 2 University of Toronto, Toronto, Canada; 3 Weatherall Institute of Molecular Medicine, Oxford, United Kingdom; 4 Centre for Infections, Health Protection Agency, London, United Kingdom; University of Cape Town, South Africa

## Abstract

**Background:**

Chronic immune activation is a hallmark of HIV infection and has been associated with disease progression. Assessment of soluble biomarkers indicating immune activation provide clues into pathogenesis and hold promise for the development of point-of-care monitoring of HIV in resource-poor-settings. Their evaluation in cohort resources is therefore needed to further their development and use in HIV research.

**Methodology/Principal Findings:**

Longitudinal evaluation of βeta-2 microglobulin (β-2 m), neopterin and suPAR soluble urokinase-type plasminogen activator receptor (suPAR) was performed with archived plasma samples to predict disease progression and provided the first direct comparison of levels in HIV-1 and HIV-2 infections. At least 2095 samples from 137 HIV-1 and 198 HIV-2 subjects with starting CD4% of ≥28 and median follow up of 4 years were analysed. All biomarkers were correlated negatively to CD4% and positively to viral load and to each other. Analyses in subjects living for ≥5 years revealed increases in median β-2 m and neopterin and decreases in CD4% over this period and the odds of death within 5 years were positively associated with baseline levels of β-2 m and neopterin. ROC analyses strengthened the evidence of elevation of biomarkers in patients approaching death in both HIV-1 and HIV-2 infections. Regression models showed that rates of biomarker fold change accelerated from 6–8 years before death with no significant differences between biomarker levels in HIV-1 and HIV-2 at equal time points prior to death.An ‘immune activation index’ analysis indicative of biomarker levels at equivalent viral loads also showed no differences between the two infections.

**Conclusions/Significance:**

Our results suggest that β-2 m and neopterin are useful tools for disease monitoring in both HIV-1 and HIV-2 infections, whereas sUPAR performed less well. Levels of immune activation per amount of virus were comparable in HIV-1 and HIV-2 infected subjects.

## Introduction

Chronic immune activation, a hallmark of HIV-1 infection [1], has been associated with disease progression [2], [3] and has even been put forward as a stronger predictive factor for progression than CD4 T cell count or viral load [4], [5]. Massive CD4 T cell depletion in HIV-1 infection can be accounted for by a direct effect of the virus on gut lymphoid tissue during initial stages of infection [6] and this is associated with aberrant immune activation [7]. Increased immune activation has also been reported during HIV-2 infection, but it is still unclear whether it is of similar importance to that observed in HIV-1 infected subjects [3], [8]. Observations in the sooty mangabey model of simian immunodeficiency virus (SIV) infection from which HIV-2 is derived shows that animals generally remain healthy despite high plasma viral loads but there is a striking absence of generalised T cell activation [9]. By contrast, SIV infected macaques, which develop disease and die rapidly, have high levels of immune activation. Furthermore, the few human subjects who develop AIDS among HIV-1 positive elite controllers (with undetectable plasma HIV RNA levels) have abnormally high T cell activation which is thought to contribute to their progressive CD4 T cell loss in the absence of viraemia [10]. These all suggest a causal link between generalised activation of the immune system and progressive immunodeficiency.

**Table 1:** Baseline characteristics of all subjects recruited into the study with comparison of HIV-1 vs. HIV-2 patients.

	Femalen (%)	Malen (%)	Age (years)(IQR)	CD4%(IQR)	β2 m (mg/L)(IQR)	Neopterin (nmol/L) (IQR)	sUPAR (µg/L)(IQR)
**HIV-1 (n = 137)**	105 (77%)	32 (23%)	29 (25–35)	34 (30–39)	2.05 (1.69–2.84)	11.6 (8.0–20.0)	3.72 (2.92–4.75)
**HIV-2 (n = 198)**	160 (81%)	38 (19%)	32 (27–41)	34 (31–40)	1.75 (1.43–2.57)	10.3 (7.3–17.2)	3.85.07–5.40)
**P-value**	0.412		0.002	0.185	0.001	0.062	0.162

Inter-quartile ranges for the age, CD4% and the biomarkers are shown in brackets.

Three serum biomarkers of immune activation, namely βeta-2 microglobulin (β-2 m), neopterin and soluble urokinase-type plasminogen activator receptor (suPAR) have been shown to correlate with HIV disease progression [11]–[18]. βeta-2 m comprises the light chain of the major histocompatibility antigen (HLA) and is found on the surface of most nucleated cells, with particular abundance on the surface of lymphocytes. Neopterin is a catabolic product of Guanine Tri Phosphate (GTP) synthesised by macrophages upon stimulation with interferon-gamma, and thus reflects the degree of Th1-type immune activation. sUPAR is the soluble form of the urokinase-type plasminogen activator receptor (uPAR or CD87), a glycoprotein receptor mainly expressed on immune cells, including neutrophils, activated T-cells, and macrophages [19], and can be cleaved and shed into the plasma. All three biomarkers reflect heightened immune activation, and plasma/serum levels have been shown significantly elevated in advanced HIV-1 disease (symptomatic AIDS) compared to the asymptomatic phase of infection and positively correlate with viral load and negatively correlate with CD4 count [11]–[12]. Furthermore, initiation of anti-retroviral therapy in AIDS patients showed a decrease in the biomarker levels similar to changes in viral load and CD4 counts [12]–[13], [20] and specific biomarker levels have been demonstrated in various studies to be independently predictive of progression or development of AIDS [11], [14]–[17], [21].

To date, there is no developed cheap point-of-care monitoring tool for HIV progression. The rapidly measurable soluble biomarkers are cheaper than CD4 count monitoring and hold promise for the development of point-of-care monitoring of HIV in resource-poor-settings. Their comprehensive evaluation in a longitudinal cohort resource is therefore needed in order to estimate the prospect of their development and use in HIV research. Evaluation of these biomarkers in HIV cohorts [12], [18], [21], [22] including our initial work [23]–[24] has been limited by inadequate prospective head-to-head comparisons of both HIV types and/or restricted by disease stage and suboptimal assays. With current improvement of the soluble biomarker assays, we examined a well characterised long standing (over 20 years) HIV cohort comprising HIV-1 and HIV-2 subjects coupled to a well developed database and effectively managed sample archives, to extend and confirm conclusions about the utility of these candidate prognostic markers.

Using historical plasma samples obtained from a longitudinal study of a cohort of HIV-1 and HIV-2 positive donors attending the Genito-Urinary Medicine (GUM) clinic at the MRC Unit in The Gambia, levels of neopterin, β-2 m and sUPAR were measured. This is one of the largest well characterized clinical cohorts of HIV-2 infected individuals in the world. Baseline biomarker levels were compared between patients who lived and died and changes in biomarker levels over time were studied in order to provide clues as to whether immune activation plays a role in the slower disease progression in HIV-2 compared to HIV-1 infection, whilst at the same time provide further evidence as to whether the biomarkers might be useful for clinical monitoring of disease progression in both HIV-1 and HIV-2 infections in resource poor settings. To our knowledge, this is the first head-to-head comparison of these candidate prognostic markers in HIV-1 and HIV-2 infections in a longitudinal study.

## Materials and Methods

### Study Subjects

Eligible subjects were identified from 3073 HIV infected adult patients comprising HIV-1 and/or HIV-2 infected clinical cohort in the Genito-Urinary Medicine (GUM) at the Medical Research Council (MRC) Unit in Fajara, The Gambia. This retrospective longitudinal study utilized stored sequential plasma samples and laboratory data from the cohort database. Clinical cohorts of HIV-2 infected donors are rare and samples were obtained prior to the rollout of anti-retroviral therapy (ART) in The Gambia allowing the opportunity to follow subjects through the natural clinical course of infection.

**Table 2:** Spearman rank correlations between the CD4% and CD4 absolute counts for all the samples in HIV-1 and HIV-2.

	HIV-1	HIV-2
	CD4%	CD4%
**CD4%**	1.000	1.000
**CD4 Absolute**	0.6260 (p<0.0001)	0.5756 (p<0.0001)

Correlation coefficients (r_s_) are shown for each comparison with the p-value shown in brackets below.

The aim was to identify all adults that were healthy at inclusion, prior to the onset of disease progression. Thus inclusion criteria included infection with either HIV-1 or HIV-2, ≥18 years of age, ART naïve throughout the study period with at least two follow up CD4 time points, and a starting CD4≥28%. These criteria yielded 137 HIV-1 and 198 HIV-2 infected individuals (Table 1) who were followed up until they either died, commenced ART, or when they were last seen. A total of 2095 stored samples were available for inclusion in the study. The median length of follow up for the study subjects was 4.0 years (3.6 years and 4.4 years for the HIV-1 and HIV-2 infected subjects respectively). Individual patients had between 2 and 28 samples (median 4). HIV viral load was not measured at all visits but 37 of 137 HIV-1 infected subjects (23.4%) and 62 of 198 HIV-2 subjects (30.8%) had at least one viral load measurement with a total of 159 samples available for inclusion in the study. These viral load data points were analysed with the corresponding biomarker levels from the same time point.

All subjects gave informed written consent for their samples to be stored and used for retrospective HIV studies. This study was approved by the Gambia Government/MRC Joint Ethics Committee and the London School of Hygiene and Tropical Medicine Ethics Committee.

### CD4 T Cell Count and Viral Load Measurement

Freshly collected heparinised blood (100 **µ**L) was utilised to measure CD4 counts by flow cytometry using multitest kits (FACSCalibur; BD Biosciences, New Jersey, USA). Cells were acquired using a FACScan in the early years of cohort which was supported by manual lymphocyte counts and later by FACScalibur flowcytometer (BD) and analyzed using Cell Quest Pro software (BD) according to the manufacturer’s instructions [25]–[26]. The FACSCalibur determines both the absolute CD4 cell count (in cells per mL) and the CD4 percentage (CD4 T cells as a proportion of total lymphocytes). The CD4 percentage rather than the absolute CD4 cell count was used in this study because of observer variation in the manual lymphocyte counts occurred in early data. In addition CD4 percentage is more stable than the absolute counts, which is influenced by a number of factors, such as time of day and other concurrent infections [27]–[28]. We however, found a correlation between CD4% and CD4 cell count in both HIV-1 and HIV-2 infections (Table 2).

Viral loads (VL) were measured from HIV-1/2 RNA extracted from EDTA plasma using the QIAamp viral RNA kit (Qiagen, Venlo, The Netherlands). Plasma viral load, expressed as the number of HIV-1/2 RNA copies per mL of plasma, was quantified using an in-house viral load assay [29]. The limit of detection was 100 viral RNA copies/mL of plasma and results below the level of detection were assigned an arbitrary value of 50 copies/mL for statistical analysis.

**Table 3:** Spearman rank correlations between the biomarkers, CD4% and viral loads (VL) for all samples in HIV-1 and HIV-2.

		HIV-1					HIV-2		
	**CD4%**					**CD4%**			
**CD4%**	1.000	**β2** **m**			**CD4%**	1.000	**β2** **m**		
**β2** **m**	−0.417 **(p<0.001)**(n = 749)	1.000	**Neopt**		**β2** **m**	−0.334 **(p<0.001)**(n = 1323)	1.000	**Neopt**	
**Neopterin**	−0.412 **(p<0.001)**(n = 758)	0.789 **(p<0.001)**n = 750)	1.000	**sUPAR**	**Neopterin**	−0.330 **(p<0.001)**(n = 1322)	0.767 **(p<0.001)**(n = 1313)	1.000	**sUPAR**
**sUPAR**	−0.083 **(p = 0.023)**(n = 750)	0.472 **(p<0.001)**(n = 747)	0.413 **(p<0.001)**(n = 750)	1.000	**sUPAR**	−0.162 **(p<0.001)**(n = 1323)	0.561 **(p<0.001)**(n = 1317)	0.531 **(p<0.001)**(n = 1317)	1.000
**Viral Load**	−0.424 **(p = 0.001)**(n = 54)	0.488 **(p<0.001)**(n = 54)	0.461 **(p<0.001)**(n = 54)	0.456 **(p<0.001)**(n = 54)	**Viral Load**	−0.337 **(p<0.001)**(n = 105)	0.397 **(p<0.001)**(n = 103)	0.346 **(p<0.001)**(n = 104)	0.232 **(p = 0.018)**(n = 103)

Correlation coefficients (r_s_) are shown for each comparison with the p-value and number of samples shown in brackets below.

### Plasma Beta-2 Microglobulin, Neopterin and sUPAR Assays

Plasma was collected from heparinised blood using standard methods and stored at −70°C. Baseline plasma β2 m, neopterin and sUPAR levels were measured at enrolment and longitudinally at all time points for which CD4 counts and plasma were available. The β2 m levels were assessed by an automated Microparticle Enzyme Immunoassay (MEIA) using an AxSYM automated machine (Abbott Laboratories, Wiesbaden, Germany). The neopterin assay was performed using the Neopterin Enzyme Immunoassay (B·R·A·H·M·S Diagnostica, Berlin, Germany). Plasma sUPAR was measured using a commercial sUPARnostic® ELISA assay (ViroGates, Birkerød, Denmark) which is a simplified double monoclonal antibody sandwich assay. All assays were performed according to the manufacturer’s instructions. The normal ranges for healthy HIV negative individuals are: β2 m 1.05–3.9 mg/L; neopterin 3–8 nmol/L; sUPAR 1.6–3.8 **µ**g/L.

### Statistical Analysis

Statistical analyses were performed using STATA 11.2 (StataCorp, College Station, TX). Concentrations of β2 m, neopterin and sUPAR were not Normally distributed therefore non-parametric methods were used for simple comparisons of groups whilst log transformed values were used in regression models to permit adjusting for age. Means of logged values were back transformed (antilogged) to obtain geometric mean values. Throughout this paper the term ‘stable’ is used to refer to subjects whose last CD4% was ≥28% while those whose last CD4% was <28% are referred to as ‘progressors’. For all statistical tests p-values <0.05 were considered significant.

Baseline characteristics (CD4%, β2 m, neopterin, sUPAR and age) were compared between HIV types and between sexes within HIV types using Wilcoxon ranksum tests. Sex distributions were compared using Fisher’s exact test. The correlations between CD4%, viral load and immune activation markers in all samples were evaluated using Spearmans’ rank correlation tests.

For patients who had ≥5 years of follow-up or were known to have died within 5 years of their first sample the odds of death within 5 years were compared between HIV types using logistic regression. The ability of the baseline biomarker concentrations to predict death within 5 years was investigated further with Receiver Operating Characteristic (ROC) curves. Since lower values of CD4% and higher values of the biomarkers are associated with poor prognosis, 100-CD4% (i.e. non-CD4%) was used in these analyses to allow direct comparison with the biomarkers. The areas under the curves were compared for the different biomarkers, larger areas indicating better prognostic accuracy.

For those patients who were known to be still alive after 5 years of follow-up, CD4% and biomarker concentrations at 5 years were estimated for each patient by linearly interpolating between the two nearest time points available. Wilcoxon matched pairs signed ranks tests were used to compare baseline and 5 year estimated values and the median changes (decrease in CD4% or increase in biomarker concentrations) between baseline values and 5 year estimates were compared between HIV types using Wilcoxon ranksum tests.

**Table 4:** Comparison of median baseline and the 5 year estimated values of CD4%, β2 m, neopterin and sUPAR for HIV-1 and HIV-2 infected subjects who were still alive after 5 years of follow up.

	CD4%				β2 m(mg/L)				Neopterin(nmol/L)				sUPAR(µg/L)			
	Baseline	5 year estimate	5 year change*	Baseline vs 5yr p-value**	Baseline	5 year estimate	5 year change*	Baseline vs 5yr p-value**	Baseline	5 year estimate	5 year change*	Baseline vs 5yr p-value**	Baseline	5 year estimate	5 year change*	Baseline vs 5yr p-value**
HIV-1 (n = 49)	34	25	−13	<0.001	2.28	2.58	0.27	0.124	12.3	17.3	3.7	0.018	4.16	4.43	0.04	0.834
HIV-2 (n = 91)	34	32	−3	<0.001	1.65	2.02	0.12	0.021	9.8	13.0	0.8	0.040	3.90	4.02	0.02	0.720
HIV-1 vs HIV2 P-value***			<0.001				0.767				0.383				0.733	

* Median of pairwise differences (5 year estimates – baseline values).

** Wilcoxon matched pairs signed ranks test for baseline vs 5 years.

*** Wilcoxon ranksum test comparing 5 year changes in HIV-1 patients with 5 year changes in HIV-2 patients.

For all patients who were known to have died at any time during the study, age at death was estimated using Kaplan-Meier survival curves and compared between HIV types using a logrank test. All available measurements of CD4% and the biomarkers prior to death from these patients were grouped into 1 year age bands from the time of their death backwards. Mean CD4% and geometric mean concentrations of the biomarkers at equivalent times before death were compared between HIV types and with the values in patients who were still alive and stable at the end of their follow-up (i.e. their last CD4% was ≥28%) using mixed effects linear regression, adjusting for age as a fixed covariate and patient as a random factor to allow for multiple samples from some individuals.

To examine the patterns of change of the biomarkers over time in patients approaching death three regressions models were compared with CD4% or log biomarker concentration as the dependent variable. The first model (a linear model) assumed a linear decrease or increase from the start of follow-up. The second model (a breakpoint model) assumed a constant level of the biomarker up to some point, followed by a linear change until the point of death. The third model (a non-linear model) assumed an accelerating rate of change as the patient approached death:
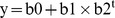
where t is the time before death, y is the concentration of the biomarker, b0 is asymptotically the stable concentration, b0+b1 is the concentration at the time of death and b2 is a parameter defining the rate of acceleration of increase or decrease in the biomarker. As log biomarker concentrations were used as the response variable (other than for CD4%) a linear trend in these models implies constant fold changes in the biomarker per unit time as patients approached death. The goodness of fit of the models were compared using adjusted R-squared values (higher is better) and Akaike's Information Criterion (AIC) (lower is better). The models were fitted for each biomarker using non-linear regression with a clustered robust variance estimator to allow for multiple samples from patients. Separate models were fitted for HIV-1 and HIV-2 patients and the coefficients compared using Wald tests.

**Figure 1:**
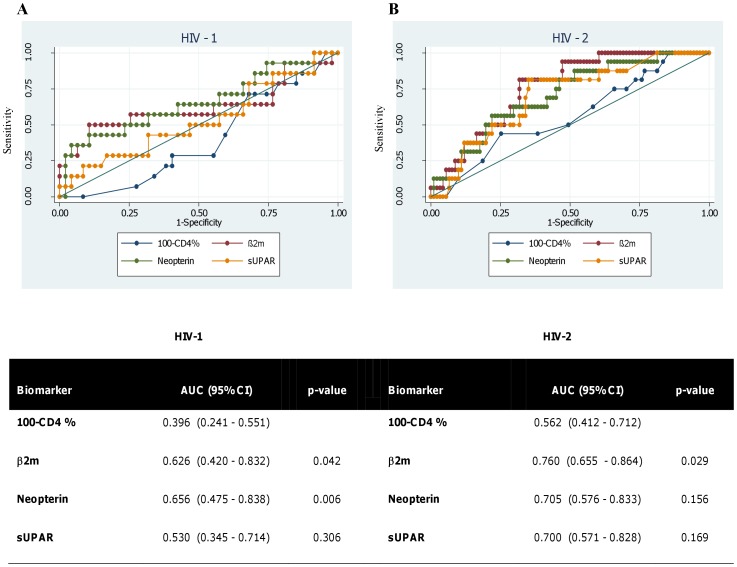
Prediction of death from baseline measurements. ROC curves for prediction of death within 5 years from baseline measurements of CD4% and the biomarkers for HIV-1 (A) and HIV-2 (B) subjects. Areas under the curves (with 95% confidence intervals) are also shown. P-values indicate significance of the difference in areas compared with the curve for 100-CD4%. Curves approaching the top left hand corner of the graph and an area under the curve (AUC) closer to 1 indicate better predictive ability. An AUC of 0.5 is equivalent to guessing and an AUC of 1 would correspond to perfect prediction of which patients who would die within 5 years and which would not.

The association between concentrations of the biomarkers and VL was investigated using all samples for which VL data were available. “Immune activation indices” were calculated for each of the biomarkers, defined as biomarker concentration divided by viral load. Mixed effects linear regression models of log immune activation index on log VL were fitted adjusting for age and including patient as a random factor to allow for multiple samples from some patients.

## Results

### Baseline Characteristics

The median initial CD4% for HIV-1 and HIV-2 infected subjects were comparable as expected since a starting CD4% >28% was a criterion for entry into the study. Though baseline values of β-2 m were within normal ranges HIV-2 subjects had significantly lower levels compared to HIV-1 infection (HIV-1 2.05 mg/L, HIV-2 1.75 mg/L, p  = 0.001). This was not the case with neopterin and sUPAR that had variable readouts and showed no differences between the two HIV subgroups (Table 1).

**Table 5:** Baseline CD4% and concentrations of the biomarkers in patients who were known to have died or known to be still alive after the first 5 years of follow-up.

	CD4%				β2 m(mg/L)				Neopterin(nmol/L)				sUPAR(µg/L)			
	HIV-1		HIV-2		HIV-1		HIV-2		HIV-1		HIV-2		HIV-1		HIV-2	
	Alive (n = 49)	Dead (n = 14)	Alive(n = 91)	Dead (n = 16)	Alive (n = 49)	Dead (n = 14)	Alive (n = 91)	Dead(n = 16)	Alive (n = 49)	Dead(n = 14)	Alive (n = 91)	Dead(n = 16)	Alive (n = 49)	Dead(n = 14)	Alive (n = 91)	Dead(n = 16)
median	34	36	34	33.5	2.28	3.20	1.65	2.64	12.3	19.7	9.8	19.0	4.16	4.08	3.90	5.35
minimum	28	29	28	29	0.96	1.16	0.75	1.55	4.0	6.8	3.6	6.5	1.00	2.60	2.00	3.00
25th percentile	29	33	31	30.5	1.78	1.74	1.32	2.15	7.9	9.2	6.9	10.8	3.30	3.40	3.00	4.68
75th percentile	39	41	38	36	2.91	5.11	2.49	3.49	20.3	50.2	17.6	28.8	5.01	5.62	5.50	7.25
maximum	60	49	61	42	5.35	9.36	5.80	15.47	108.0	101.6	151.3	141.4	17.00	43.40	19.40	8.70
p-value*	0.456		0.346		0.021		0.023		0.024		0.047		0.191		0.126	

* Effect of baseline CD4% or biomarker concentration on odds of death within 5 years using logistic regression.

**Table 6:** Mean CD4% and geometric mean biomarker levels at different times before death in patients who died during follow-up compared to levels in stable subjects who were still alive at the end of follow-up.

		HIV1				HIV2			HIV1 vs
				p-value				p-value	HIV2
years before death		n	mean/geometric mean*^*^	(compared to stable)		n	mean/geometric mean*^*^	(compared to stable)	p-value
**CD4%**									
<1		28	15.9	<0.001		32	21.6	<0.001	0.022
1 - <2		25	21.8	<0.001		33	24.3	<0.001	0.341
2 - <3		20	27.4	<0.001		23	26.7	<0.001	0.775
3 - <4		18	27.8	0.001		23	27.5	<0.001	0.907
4 - <5		16	30.3	0.021		20	31.0	0.002	0.833
5 - <6		16	31.3	0.065		24	33.3	0.034	0.493
6 - <7		14	33.0	0.302		13	36.0	0.564	0.370
7 - <8		7	38.3	0.366		17	33.8	0.098	0.198
8 - <9		4	42.3	0.080		9	38.4	0.699	0.374
stable		236	35.4			778	37.3		0.112
**β2** **m (mg/L)**									
<1	28		4.2	<0.001	32		4.2	<0.001	0.533
1 - <2	25		3.5	<0.001	33		3.3	<0.001	0.282
2 - <3	20		3.2	<0.001	23		2.8	<0.001	0.221
3 - <4	18		2.8	0.001	23		2.8	0.001	0.720
4 - <5	16		2.6	0.004	20		2.3	0.126	0.163
5 - <6	16		2.6	0.007	24		2.3	0.058	0.272
6 - <7	14		2.5	0.014	13		2.0	0.494	0.100
7 - <8	7		2.6	0.025	17		2.1	0.472	0.101
8 - <9	4		2.1	0.566	9		1.7	0.504	0.252
stable	236		1.9		778		1.9		0.359
**Neopterin (nmol/L)**									
<1	28		32.1	<0.001	32		29.8	<0.001	0.490
1 - <2	25		24.3	<0.001	33		22.5	<0.001	0.562
2 - <3	20		22.1	<0.001	23		21.5	<0.001	0.813
3 - <4	18		16.3	0.018	23		19.4	<0.001	0.425
4 - <5	16		14.9	0.062	20		17.2	0.001	0.565
5 - <6	16		14.5	0.090	24		19.6	<0.001	0.158
6 - <7	14		15.9	0.025	13		15.2	0.027	0.760
7 - <8	7		18.4	0.012	17		15.7	0.011	0.446
8 - <9	4		10.4	0.851	9		11.4	0.701	0.831
stable	236		11.0		778		10.5		0.503
**sUPAR (µg/L)**									
<1	28		5.1	0.001	32		6.7	<0.001	0.023
1 - <2	25		4.9	0.003	33		5.2	<0.001	0.619
2 - <3	20		4.8	0.007	23		4.7	0.022	0.926
3 - <4	18		4.2	0.129	23		5.1	0.003	0.148
4 - <5	16		4.3	0.086	20		4.4	0.225	0.932
5 - <6	16		4.0	0.286	24		4.7	0.027	0.187
6 - <7	14		4.7	0.014	13		4.5	0.125	0.776
7 - <8	7		5.4	0.002	17		4.8	0.022	0.380
8 - <9	4		4.7	0.073	9		4.4	0.311	0.672
stable	236		3.5		778		3.9		0.071

Comparisons between HIV-1 and HIV-2 patients at similar times before death are also shown. All p-values are adjusted for age of the patient.

### Biomarker Correlations with CD4% and VL in HIV-1 and HIV-2 Infections

A negative correlation between all three biomarkers and the CD4% was observed in both HIV-1 and HIV-2 infections (Tables 3). As expected, viral load was positively correlated with the biomarker levels and negatively correlated with CD4%, all correlations being moderate but significant. The three biomarkers correlated positively with one another in both HIV-1 and HIV-2 infected patients, with the correlation between β2 m and neopterin being the strongest (HIV-1: r_s_ = 0.789, p<0.001; HIV-2: r_s_ = 0.767, p<0.001). These correlations suggest that changes in these biomarkers are likely to reflect changes in CD4% and viral replication during HIV disease progression in both HIV-1 and HIV-2 infections. We therefore investigated whether the biomarkers would be useful indicators of disease progression and/or prognostic for disease outcome.

**Figure 2:**
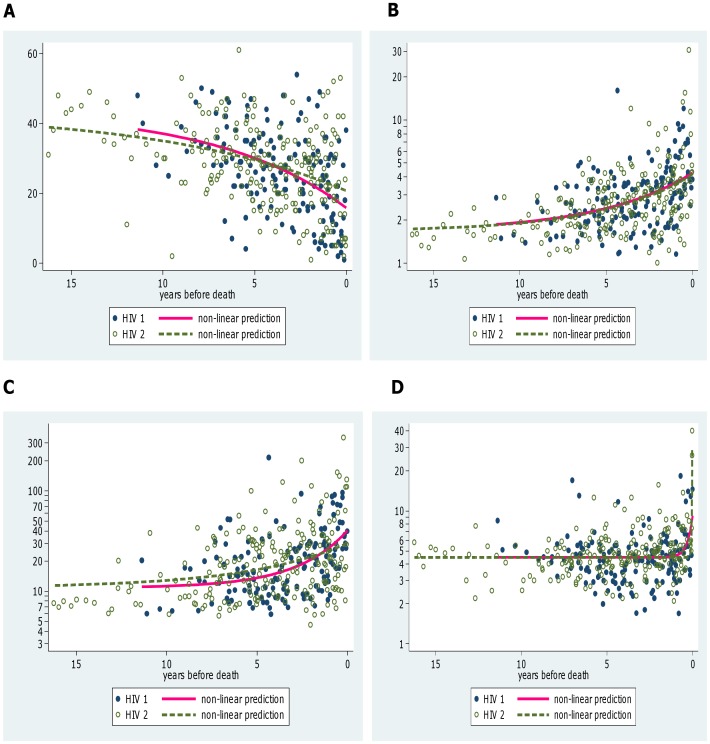
Changes in biomarkers over time. Non-linear regressions showing changes in biomarkers over time as subjects approach death in HIV-1 and HIV-2 infected individuals who died during the study for CD4% (A), β2 m (B), Neopterin (C) and suPAR (D).

### Biomarkers as Predictors of Disease Progression and Death

Since the subjects in our study were followed up for different lengths of time, we chose to compare outcomes 5 years after the first measurement in the study. Sixty three HIV-1 and 107 HIV-2 patients had ≥5 years of follow-up or were known to have died within 5 years of their first sample. Of these 14 (22%) HIV-1 and 16 (15%) HIV-2 died within 5 years. Adjusted for age there were significantly lower odds of death within 5 years in the HIV-2 group (p = 0.036) (Odds ratio [OR] = 0.38, 95% CI 0.15, 0.94). In patients who were still alive at 5 years there were significant decreases in CD4% and an increasing trend for biomarker values from baseline (Table 4). Consistent with the higher mortality rate in HIV-1 patients CD4% declined more in the live HIV-1 than HIV-2 patients over the 5 years (p<0.001), suggesting that disease progression was generally faster in the HIV-1 group from the time of their first sample. The biomarkers also showed greater median changes (increases) in HIV-1 than HIV-2 patients but the differences were not significant.

**Figure 3:**
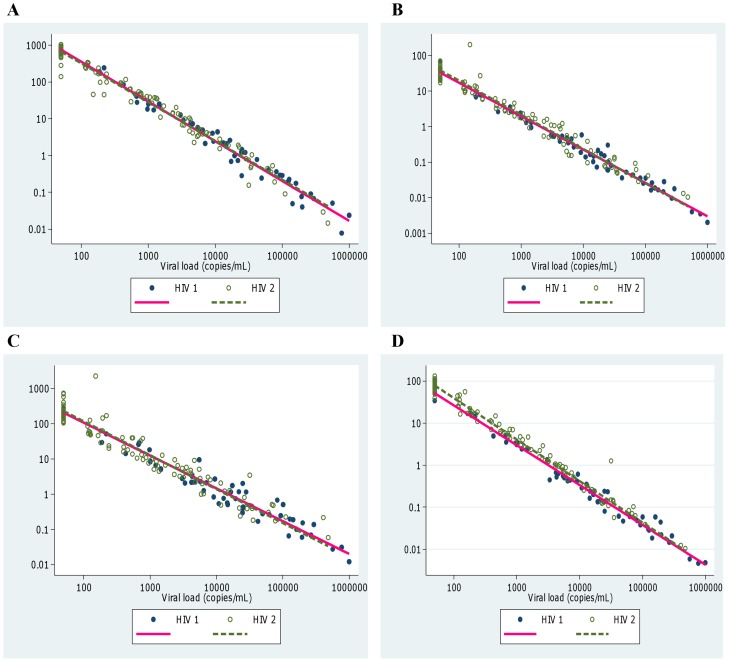
Changes in relative CD4% and immune activation index with viral load. Immune activation index is plotted on the vertical axis and defined here as concentration of the biomarker divided by viral load. CD4% per unit viral load is also plotted for comparison. (A) CD4%/viral load (copies/mL), (B) β2 m (mg/L)/viral load (copies/mL), (C) Neopterin (nmol/L)/viral load (copies/mL), and (D) suPAR (**µ**/L)/viral load (copies/mL).

The odds of death within 5 years increased significantly with increasing baseline concentrations of β2 m and neopterin in both HIV-1 and HIV-2 patients (Table 5). sUPAR showed the same trend for HIV-2 but this was not significant. In order to investigate whether there were threshold levels of the biomarkers predictive of death within 5 years ROC analyses were carried out (Figure 1). ROC analyses compare sensitivity and specificity of a prediction for different cut-off values of the predictor. An area under the curve (AUC) equal to 0.5 is equivalent to what would be achieved by guessing. As might be expected in this cohort of patients who were selected on the basis of having an initial CD4≥28%, CD4% was not indicative of the outcome at 5 years. All of the three biomarkers had AUC greater than 0.5 and this was particularly clear in the HIV-2 group where AUC was around 0.7 and all of the 95% confidence intervals were above 0.5 (i.e. using these biomarkers to predict which patients would have died would have been significantly better than guessing). The significant differences compared with the curves for CD4% suggest that these biomarkers may give better, or at least additional, information about disease progression compared with CD4% alone in similar cohorts of patients.

### No Difference in Biomarker Levels at the Same Stage of Disease Progression in HIV-1 and HIV-2 Infections

Twenty nine HIV-1 and 36 HIV-2 patients died during follow-up. Kaplan-Meier estimates of median age at death in the two diseases (which allows for censoring due to the limited follow-up times) were 56 and 58 years for HIV-1 and HIV-2 respectively (p = 0.029). However median ages at death amongst those who died during the study were 32 years (IQR 31–42) for the HIV-1 patients and 48 years (IQR 37–55) for the HIV-2 patients (p<0.001). Mean CD4% and geometric mean biomarker concentrations during successive 1 year time intervals before death for these patients are shown in Table 6. There were no differences between biomarker levels in HIV-1 compared to HIV-2 infections at equal time points prior to death (adjusted for age), except sUPAR levels that were higher in HIV-2 compared to HIV-1 infected individuals in the last year of life (p  = 0.023). In the last year of life, mean CD4% for HIV-2 infected subjects were significantly higher compared to HIV-1 infected subjects. Thus HIV-2 subjects probably died at higher CD4% compared to HIV-1 infected subjects. Concentrations in dying patients were compared with those in patients who remained alive and stable at the end of the study (last CD4% ≥28%). Significant decreases in CD4% were observed from around 5–6 years before death and significant increases in all three of the biomarkers from around 6–8 years before death in both diseases (Table 6).

### The Rate of Change of the Biomarkers Accelerates as HIV Subjects near their Death

Regression models were used to examine whether the rate of change of CD4% and the rate of fold change in the biomarkers was constant or accelerating in patients approaching death. The R^2^ values for all of the models were <0.21 and differences between the models were small. However the non-linear models gave lower AIC and higher adjusted R^2^ compared with the linear or breakpoint models for CD4% and all three of the biomarkers suggesting that rates of changes in immune activation accelerate as the disease progresses. Separate equations were fitted for HIV-1 and HIV-2 patients (Figure 2A, B, C, D) but there were no significant differences between the curves for the two diseases for CD4%, β2 m or neopterin. The estimate for sUPAR levels at the time of death was significantly higher in the HIV-2 patients which appears to be related to a small number of very high values in patients very shortly before they died (Figure 2D). The results from the non-linear regressions are consistent with the results in Table 6 showing increases from between 5 and 10 years prior to death and similar levels of immune activation in HIV-1 and HIV-2 at equivalent times.

### Higher Viral Loads Associated with Higher Immune Activation but Lower Biomarker to VL Ratio in both HIV-1 and HIV-2 Infections

The associations between concentrations of the biomarkers and VL were investigated using the 159 samples for which VL data were available. There were significant positive correlations between the biomarkers and viral load (Table 3) but we also investigated whether the concentration of the biomarkers per unit viral load (“immune activation index”) changed with increasing viral load. There were clear decreases in the immune activation indices for all three biomarkers with increasing VL (p<0.001) (Figure 3) and there were no significant differences between the lines fitted for HIV-1 and HIV-2 infected subjects for β-2 m or neopterin, suggesting similar relative levels of immune activation at equivalent viral loads in the two diseases. Immune activation indices for sUPAR were slightly higher in the HIV-2 patients (p = 0.002; Fig 3D).

## Discussion

This study shows that all three biomarkers, β2-m, neopterin and sUPAR, were significantly elevated 6-8 years before patients died compared with the patients who survived; this applied to both HIV-1 and HIV-2 infected subjects. Therefore an increase in biomarker concentrations appeared to be associated with disease progression around the same time as a decrease in CD4% was observed. The HIV subjects with higher levels of β-2 m and neopterin at baseline had higher odds of dying within 5 years in both the HIV-1 and HIV-2 groups further supporting the potential value of these biomarkers in disease monitoring in HIV infected individuals. Among those that lived there were increases in biomarker levels over 5 years in both HIV groups. Previous studies in Caio, Guinea Bissau, similarly found that β2-m predicted mortality in HIV-2 infection, followed by neopterin, whereas sUPAR did not [23], [30].

βeta-2 m and neopterin positively correlated well with viral load and inversely with CD4%, whereas sUPAR correlated less well. Non-linear regression models demonstrated that the biomarkers reflected disease progression in both HIV-1 and HIV-2 infected patients. Furthermore, the rate of change of biomarkers accelerated as death approached in both diseases, in keeping with the clinical course in the final years of life. Recent studies suggest similar immune activation in HIV-1 and HIV-2 at the same stage of disease [31], [32], and the results described herein would generally support this.

Whilst these data suggest the biomarkers have utility for monitoring progression in both diseases, and behave similarly as disease progresses, it was still not clear what role the amount of replicating virus might play. The viral load set point is generally much lower in HIV-2 than in HIV-1 infection [33]. However a proportion of HIV-2 subjects have a high set point and eventually lose viral control and progress to AIDS in a similar manner to HIV-1 infected subjects [24], [34]. Thus the differences in the rate of progression of these two infections seem to lie in the control of viral replication, which itself drives immune activation. We established an “immune activation index” which took into account the VL value for each biomarker measurement. Interestingly, this index was higher at low VLs suggesting that immune activation in these patients was more efficient at containing viral replication than those with a high viral load who had a low index. The index was higher in early disease and declined as viral control was lost in a similar way in both HIV-1 and HIV-2 infections. Both types of HIV infected individuals behaved similarly for β2 m and neopterin, further supporting similar immune activation in the 2 diseases, although the sUPAR index was higher in HIV-2 infected subjects. This observation refutes previous studies suggesting that immune activation is higher in HIV-1 infected donors [3], [8], [35] and this is probably because these studies did not allow for VL. The presence of a higher “immune activation index” in early disease further supports the potential utility of this biomarker for predicting outcome in both diseases. Since HIV-2 infected individuals retain viral control with undetectable VLs for longer [24], [29], [33], they would therefore be expected to have higher immune activation index for longer. It is thus probable that the HIV-1 who would have had consistent failed viral control with attendant higher levels of immune activation markers (giving lower index) were missed out during selection due to their inconsistent or non-follow up in the cohort. It should be borne in mind that VL data were only available for a subset of the study donors and levels were not measured at each time point, particularly in individuals that were well or stable. Adjustment for age, multiple samples and VL was performed to overcome this limitation. However further studies are required to investigate how immune activation behaves in relation to VL in the two diseases, particularly in the early asymptomatic stage when VL is under control in HIV-2 infected individuals.

Overall viral control in HIV-2 infection seems to be more clearly related to the host immune response than in HIV-1 infection [36], [37]. The relationship between immune activation, viral control and specific immune responses is clearly an important area of future research if we are to understand why HIV-2 disease progresses more slowly. The sooty mangabey primate model in which the absence of immune activation in the face of high viral loads appears to explain the lack of pathogenicity could be further exploited in our quest to understand slow progression and the pathogenesis of HIV-2. Indeed, therapeutic measures aimed at suppressing viral replication and immune activation in early HIV-1 infection have been shown to slow disease progression [38], and similar strategies might prevent progression in HIV-2.

There are certain limitations to this study including the fact that the cause of death was not known. The subjects were followed for different lengths of time, and although all of them had CD4≥28% at entry we do not know how long they had been living with HIV, although the baseline age differences would suggest that the HIV-2 subjects had been infected for longer. It should also be borne in mind that this is a clinical cohort, which is quite different to a community cohort where the HIV-2 infected individuals would be expected to present much later than the HIV-1 infected group.

In summary, the immune activation biomarkers β2 m, neopterin and sUPAR are as good as CD4% in predicting mortality in both HIV-1 and HIV-2 infections in patients who do not yet show signs of disease progression (i.e. CD4% ≥28%), although β2 m and neopterin performed better than sUPAR. They might provide a useful and cheap monitoring tool in resource poor settings. Immune activation levels were generally comparable in HIV-1 and HIV-2 infected donors in our study, even when taking viral load into account, particularly in those with progressive disease. This suggests that the mechanism of progression is similar in both diseases, but further studies are required into early viral control in stable HIV-2 infection, since understanding this is likely to provide the key to how survival might be improved in both HIV-1 and HIV-2 infected individuals.
